# Correspondence

**Published:** 1866-11

**Authors:** 


It is with pleasure that we insert the following correspondence:

CHICAGO, Oct. 4th, 1866.

DEAR SIR,—The students of Rush Medical College, wishing to preserve for their own benefit and gratification your highly instructive and interesting Address, delivered before them at the opening of the present term, have appointed a committee to request the same of you for publication. Therefore, we, the undersigned, as such committee, and on behalf of our fellow students, do most respectfully request that you will do them the honor to present them the manuscript of your Introductory Address for the purpose above mentioned.

Very Respectfully,

E. VAN BUREN,

J. A. GROESBECK,

T. B. SPALDING,

} *Committee.*

*To* J. W. FREER, M. D., *Prof. Physiology and Surg. Pathology, Rush Medical College, Chicago, Ill.*

---

*To* Messrs. E. VAN BUREN,

J. A. GROESBECK,

T. B. SPALDING,

} *Committee:*

GENTLEMEN,—I have the honor to acknowledge the receipt of your note of the 30th inst., requesting a copy of my Introductory Lecture to the Session of 1866–67, in Rush Medical College. I herewith transmit to you said copy, with many acknowledgments for the flattering terms in which you have seen fit to characterize its merits.

Most Respectfully,
Your Obed't Serv't,
J. W. FREER.

